# Aortopulmonary homograft: Extended “commando” concept for prosthetic root endocarditis with anterior septal involvement

**DOI:** 10.1016/j.xjtc.2025.05.026

**Published:** 2025-06-10

**Authors:** Giulia Agostini, Marco Pocar, Michele William La Torre, Stefano Salizzoni, Mauro Rinaldi

**Affiliations:** a Department of Surgical Sciences, University of Turin, Turin, Italy; b Division of Cardiac Surgery, Cardiovascular and Thoracic Department, ‘Città della Salute e della Scienza’ University Hospital, Turin, Italy

**Figure:**
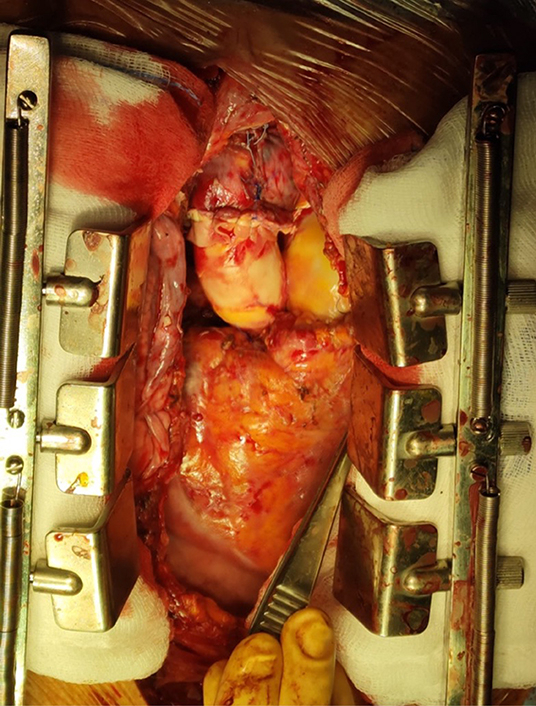
Aortopulmonary homograft for eradication of root endocarditis with anteroseptal extension.

**Central Message**

Double aortic and pulmonary homograft may be a promising approach for destructive prosthetic aortic root endocarditis with anterior septal abscess.

## Case Report

A 53-year-old man was referred 20 years after undergoing valve-sparing aortic root replacement with a modified David reimplantation technique and a 28-mm Dacron graft (Gelweave Valsalva; Terumo Vascutek) at another institution. The operation also included mitral valve posterior annuloplasty with a 34-mm Cosgrove band (Edwards Lifesciences). Recovery and early follow-up had been uneventful. Risk factors included hypertension, dyslipidemia, and a family history of emergent surgery for type A acute aortic dissection (*SMAD3* gene mutation,^1^ associated with early aortic aneurysm and dissection).

**Figure 1:**
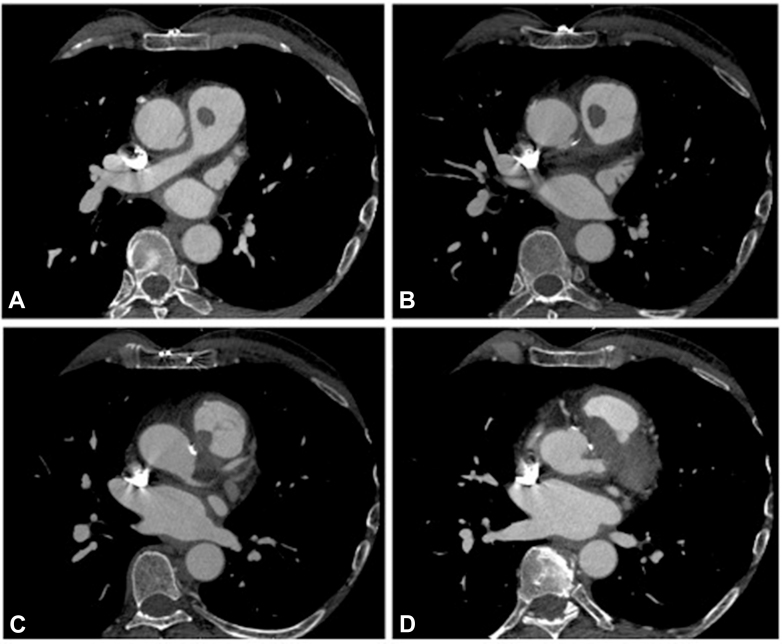
Craniocaudal sequence of 4 (A-D) axial contrast enhanced computed tomograms showing the perivalvular abscess between the great vessel roots and a 12 × 8-mm mass attached to the right pulmonary valve cusp. Note the proximity of the left main coronary artery posterior to the infected area.

After returning from a trip to India, the patient presented at the emergency department for relapsing fever, weight loss, and fatigue. Echocardiography was consistent with infective endocarditis, periprosthetic involvement, severe aortic regurgitation, and mild pulmonary valve insufficiency (Videos 1 and 2). Computed tomography confirmed an abscess involving the Dacron graft and adjacent upper interventricular septum, and also highlighted a large round mass attached to the right pulmonary valve cusp (Figure 1). Although shunting was not evident with available imaging, a fistula was suspected. Initial blood sampling was positive for methicillin-resistant *Staphylococcus epidermidis*; however, 4 subsequent four blood culture sets identified a different and unusual multiresistant *Staphylococcus*—*S. caprae*—dictating modification of the initial intravenous antibiotic regimen. The patient provided written informed consent for publication of study data. Institutional Review Board approval was not applicable.

## Technique

**Figure 2:**
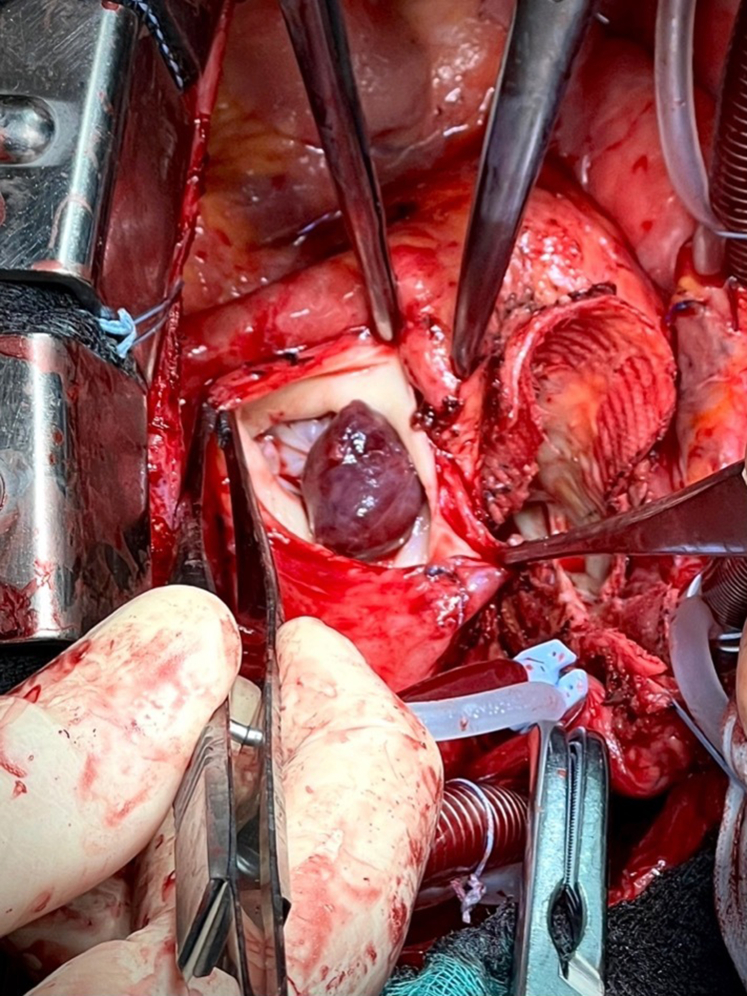
Large pulmonary valve abscess with clots as a secondary extension, aortic prosthetic endocarditis, and aortopulmonary fistula.

An unconventional operation comprising aortic and pulmonary homograft implantation was planned to treat the extensive cardiac infection with double outflow tract involvement while avoiding additional prosthetic material. After explantation of the vascular prosthesis and excision of aortic valve cusps, the precise extension of the infectious process (microbiologically analyzed as vegetation) was identified inferior to the intercommissural triangle between the left and right coronary cusps, with deep erosion into the interventricular septum and a fistulous communication with the right ventricular outflow tract. A large thrombosed vegetation (microbiologically analyzed as abscess with clots) originating from the fistula was detected on the pulmonary side and excised (Figure 2). Infected tissues were extensively debrided, along with topical disinfection with iodopovidone and 0.6% glutaraldehyde solution.^2^

A left atriotomy was performed, but the mitral ring was completely endothelialized and not involved by the infectious process, and thus it was left intact.

**Figure 3:**
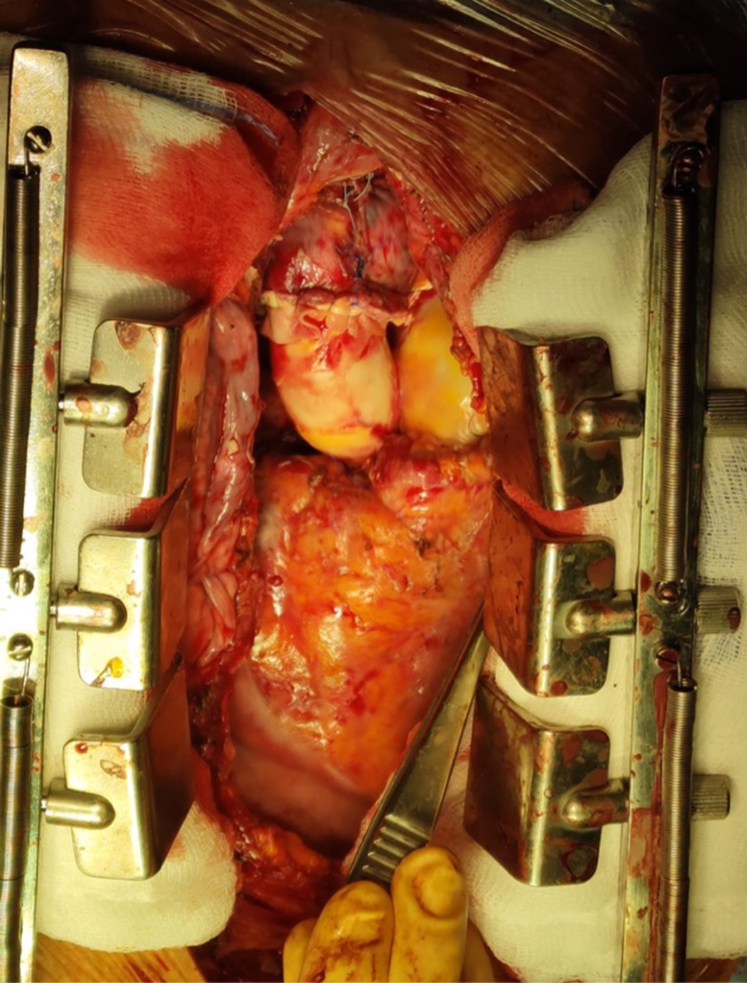
Final aspect of double outflow tract homograft reconstruction with direct attachment to the interventricular septum and right ventricle.

**Figure 4:**
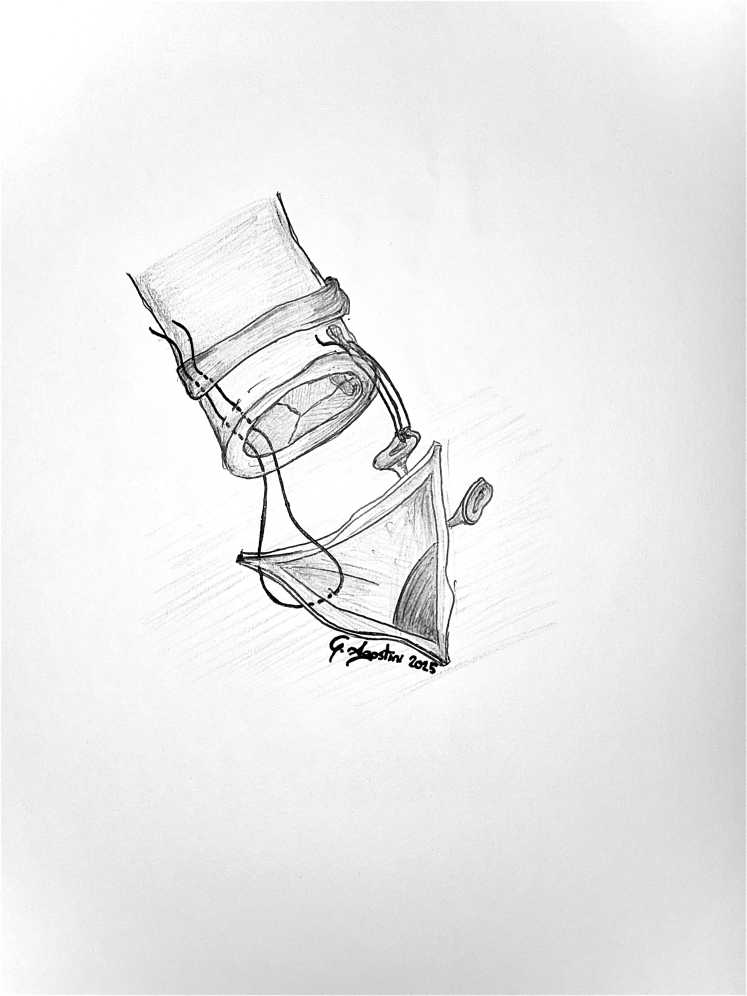
Graphic representation of the aortic homograft implantation technique.

A 23-mm aortic homograft was implanted as a full root with a button-Bentall technique and secured proximally to the left ventricular outflow tract with multiple interrupted nonpledgeted U-shaped 3/0 polypropylene sutures proximal to the ventricular outflow tract.^3^ The coronary ostia were reimplanted with 5-0 polypropylene running suture. Subsequently, a 27-mm pulmonary homograft was implanted with continuous 4-0 polypropylene sutures distally in the distal pulmonary trunk, proximally at the level of the ventricular outflow tract (Figure 3). The distal anastomosis of the aortic homograft 4-0 polypropylene continuous suture reinforced with an external bovine pericardial strip completed the reconstruction (Figure 4).

The patient's course was free of complications, and he was discharged to a rehabilitation facility on postoperative day 5. At a 6-month follow-up, echocardiography showed normal aortic, mitral, and pulmonary valve function with no intracardiac shunt, pseudoaneurysm, or sign of relapsing infection.

## Discussion

Reoperative radical surgery for destructive aortic root endocarditis with involvement of prosthetic material and adjacent structures is a challenging and high-risk scenario. Homografts might not always be readily available but represent an ideal substitute for ventricular outflow reconstruction in terms of tissue compliance and absence of additional foreign material.^3^ Staphylococci, often multiresistant, are among the most common agents in this context; however, *S. caprae* has been reported in only 5 cases of endocarditis, including perivalvular abscess.^4^

Most often, the latter involves the subaortic curtain. At this level, the so-called “commando” procedures rely on the concept that radical excision of infected tissues implies replacement and reconstruction of the aortomitral junction, preferably preserving the mitral apparatus (hemi-commando).^5^ Thus, the neoaortic root is anchored to the anterior native or prosthetic mitral (neo)annulus, avoiding patch reconstruction of the subaortic curtain and the consequent hemodynamic systolic stress and hazard of subsequent dehiscence, which potentially is enhanced by stiffer prosthetic material.

When, less commonly, root endocarditis extends to anterior perivalvular structures, the current approach typically implies pericardial patch implantation for septal outflow reconstruction. In the present case, a true ventricular septal defect had not completely developed, but substantial muscle debridement was mandatory for IE eradication. With the approach described here, both homografts were anchored directly to the ventricular outflow tracts, applying the “commando” philosophy to avoid direct septal reconstruction.

## Conclusions

Double aortopulmonary homografts may represent a promising option to eradicate prosthetic root endocarditis with anterior extension to the interventricular septum. More proximal anchoring to the ventricular outflow tracts with excess homograft tissue may promote a stable reconstruction by avoiding direct patch reconstruction of debrided and fragile septal muscle.

## Conflict of Interest Statement

The authors reported no conflicts of interest.

The *Journal* policy requires editors and reviewers to disclose conflicts of interest and to decline handling or reviewing manuscripts for which they may have a conflict of interest. The editors and reviewers of this article have no conflicts of interest.

## Supplementary Data

Video 1 Parasternal short axis view showing the perivalvular abscess, associated aortic regurgitation and the vegetation/thrombus. Video available at: https://www.jtcvs.org/article/S2666-2507(25)00247-0/fulltext.

Video 2 Long axis and short axis views showing the aortic regurgitation and the vegetation/thrombus. Video available at: https://www.jtcvs.org/article/S2666-2507(25)00247-0/fulltext.
