# Supplementary figures and images for: Aortopulmonary homograft: Extended “commando” concept for prosthetic root endocarditis with anterior septal involvement
Source: JTCVS Tech. 2025 Jun 10;32:76–80. doi: 10.1016/j.xjtc.2025.05.026 (PMC12347684; doi:10.1016/j.xjtc.2025.05.026)

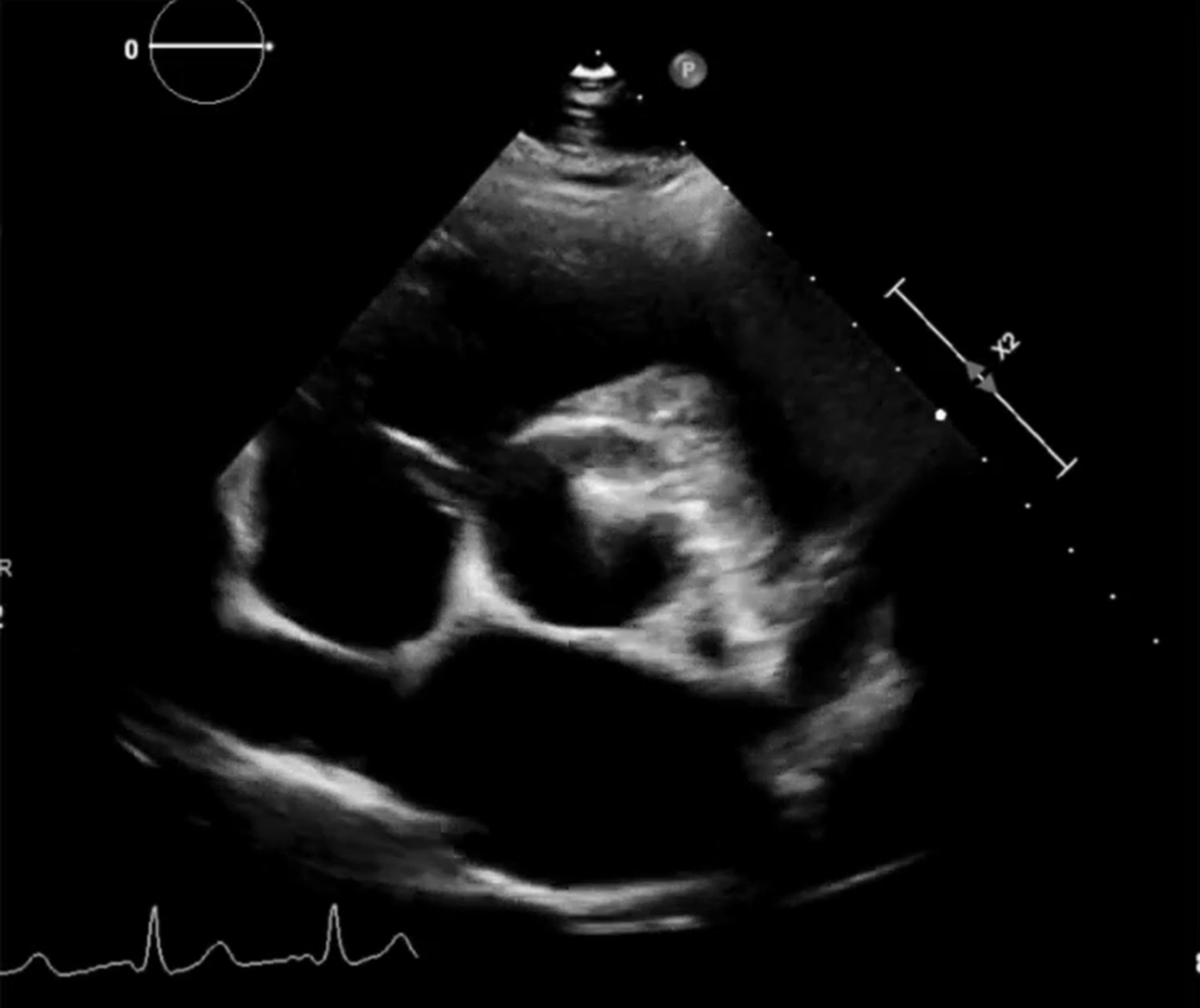

Supplement: Video 1 — Parasternal short axis view showing the perivalvular abscess, associated aortic regurgitation and the vegetation/thrombus. Video available at: https://www.jtcvs.org/article/S2666-2507(25)00247-0/fulltext. [file fx2.jpg]

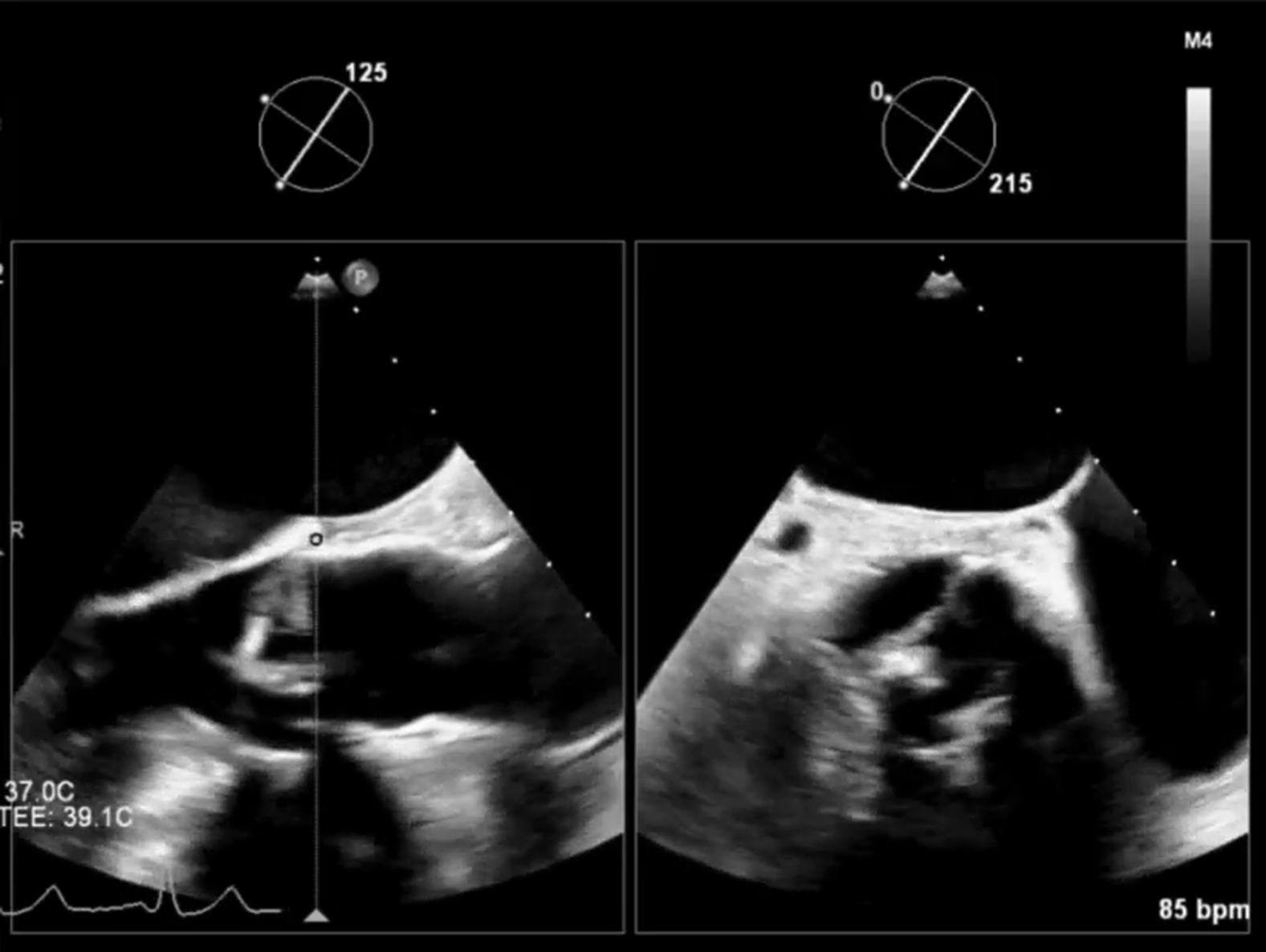

Supplement: Video 2 — Long axis and short axis views showing the aortic regurgitation and the vegetation/thrombus. Video available at: https://www.jtcvs.org/article/S2666-2507(25)00247-0/fulltext. [file fx3.jpg]
